# Crystal structure of di­aqua­bis­(2-chloro­pyridine-κ*N*)bis­(thio­cyanato-κ*N*)nickel(II)

**DOI:** 10.1107/S2056989016015218

**Published:** 2016-09-30

**Authors:** Stefan Suckert, Inke Jess, Christian Näther

**Affiliations:** a Institut für Anorganische Chemie, Christian-Albrechts-Universität Kiel, Max-Eyth Str. 2, D-24118 Kiel, Germany

**Keywords:** crystal structure, discrete metal complex, nickel(II) thio­cyanate, 2-chloro­pyridine, hydrogen bonding

## Abstract

The asymmetric unit of the title compound, [Ni(NCS)_2_(C_5_H_4_ClN)_2_(H_2_O)_2_], consists of one nickel(II) cation that is located on a center of inversion and one thio­cyanate anion, one water mol­ecule and one 2-chloro­pyridine ligand all occupying general positions. The Ni^II^ cation is octa­hedrally coordinated by two terminal N-bound thio­cyanato ligands, two aqua ligands and two N-bound 2-chloro­pyridine ligands into discrete complexes. Individual complexes are linked by inter­molecular O—H⋯S and O—H⋯Cl hydrogen-bonding inter­actions into a layered network extending parallel to the *bc* plane. Weak inter­actions of types C—H⋯S and C—H⋯Cl consolidate the crystal packing.

## Abstract 2

The crystal structure of the title compound consists of discrete octa­hedral complexes that are linked by inter­molecular O—H⋯S, C—H⋯Cl, C—H⋯S and C—H⋯Cl hydrogen bonding.

## Chemical context   

The synthesis of materials with inter­esting cooperative magnetic properties is still a major field in coordination chemistry (Zhang *et al.*, 2011 ▸). One feasible strategy for the preparation of such compounds is to link paramagnetic cations with small anionic ligands such as, for example, thio­cyanate anions to enable a magnetic exchange between the cations (Palion-Gazda *et al.*, 2015 ▸; Massoud *et al.*, 2013 ▸). In this regard, our group has reported on a number of coordination polymers with bridging thio­cyanato ligands. Dependent on the metal cation and the neutral co-ligand, they show different magnetic phenomena including a slow relaxation of the magnetization, which is indicative for single-chain magnetism (Werner *et al.*, 2014 ▸, 2015*a* ▸,*b* ▸,*c* ▸). In the context of this research, discrete complexes are likewise of inter­est because such compounds can be transformed into the desired polymeric systems by thermal decomposition (Näther *et al.*, 2013 ▸). In view of our systematic studies, we became inter­ested into compounds based on 2-chloro­pyridine as co-ligand, for which only two different polymorphs were found for representatives containing Zn or Co (Wöhlert *et al.*, 2013 ▸). In a more recent study, investigations were also carried out for Ni that led to the title compound being characterized by single crystal X-ray diffraction. Unfortunately, no single-phase crystalline powder could be synthesized, which prevented further investigations of its physical properties.

## Structural commentary   

**Figure 1:**
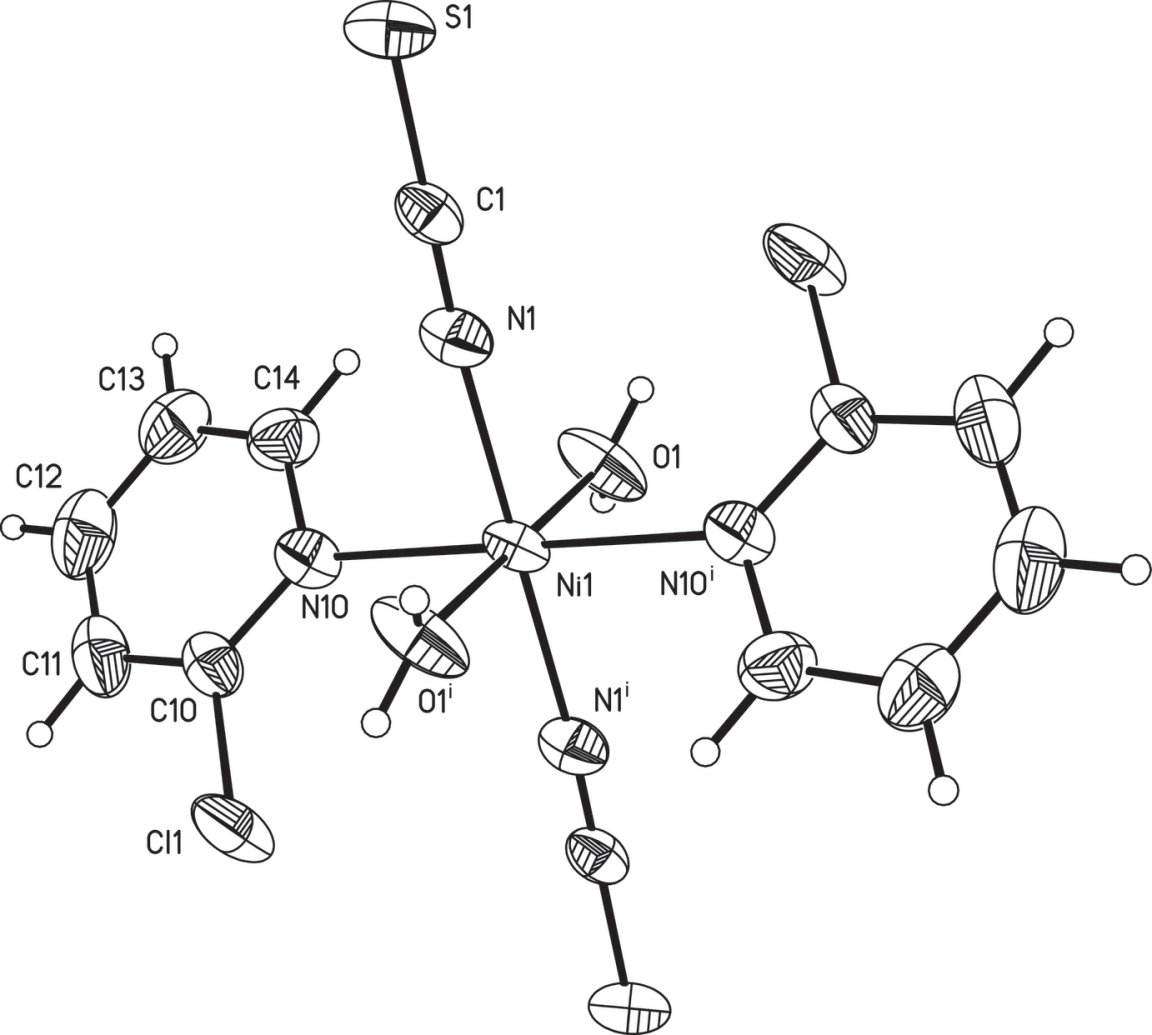
View of a discrete complex with labelling and displacement ellipsoids drawn at the 50% probability level. [Symmetry code: (i) −*x* + 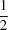, −*y* + 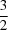, -*z.*+1.]

The asymmetric unit of the title compound, [Ni(NCS)_2_(C_5_H_4_NCl)_2_(H_2_O)_2_], consists of one Ni^II^ cation, one thio­cyanate anion, one water mol­ecule and one neutral 2-chloro­pyridine co-ligand. The cation is located on a center of inversion whereas all ligands are located on general positions. The Ni^II^ cation is coordinated by two terminal N-bound inorganic anionic ligands, two water mol­ecules and two 2-chloro­pyridine ligands that are coordinated *via* the pyridine N atom in an all-*trans* configuration (Fig. 1 ▸). As expected, and in agreement with values reported in literature (Đaković *et al.*, 2008 ▸; Werner *et al.*, 2015*b* ▸), the Ni—N bond lengths to the thio­cyanato ligands are significantly shorter[2.018 (3) Å] than to the pyridine N atom of the neutral 2-chloro­pyridine ligand [2.208 (3) Å].
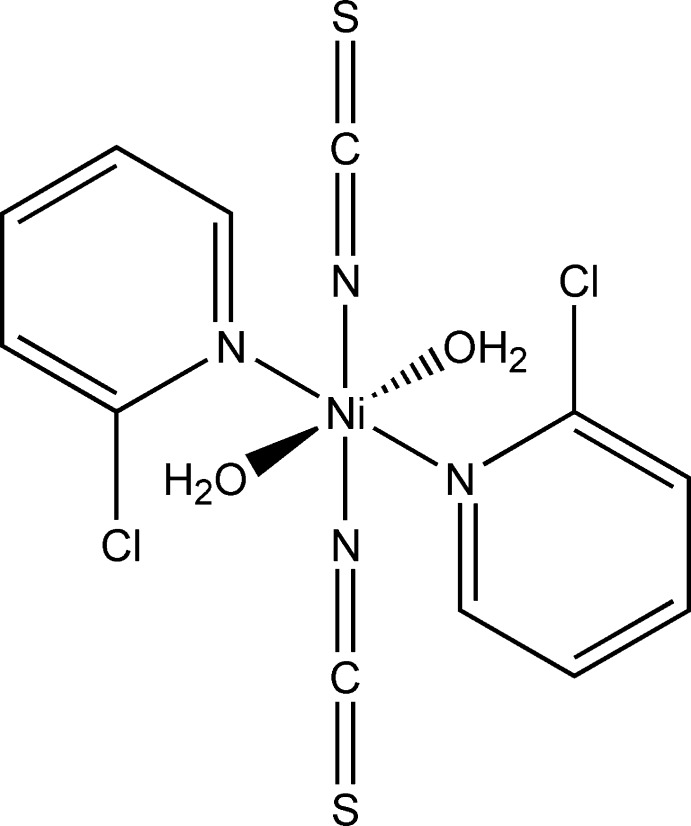


## Supra­molecular features   

**Figure 2:**
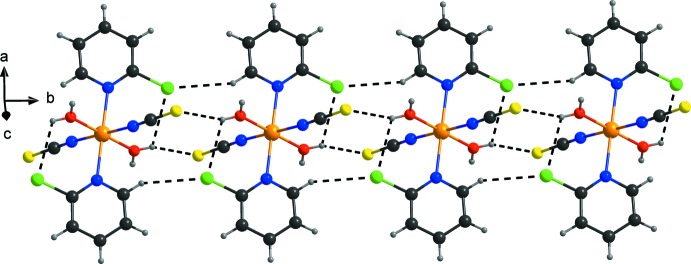
View of the hydrogen-bonded chain that elongates along the *b* axis. Hydrogen bonds are shown as dashed lines.

**Figure 3:**
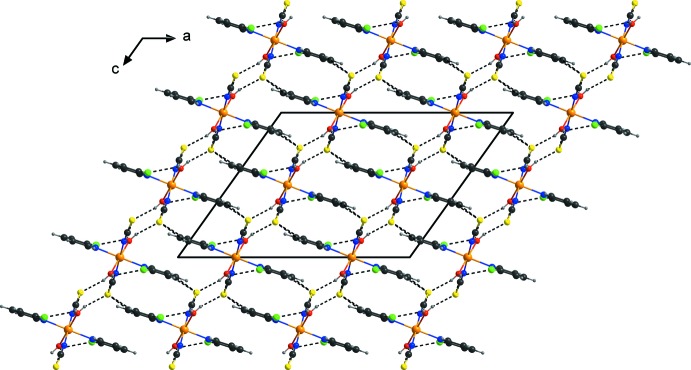
Crystal structure of the title compound showing the hydrogen-bonded layers with hydrogen bonds shown as dashed lines.

In the crystal, discrete complexes are linked by pairs of inter­molecular O—H⋯S hydrogen bonds between one of the two water H atoms and the thio­cyanato S atoms of a neighboring complex into centrosymmetric dimers that are further connected into chains along the *b* axis (Fig. 2 ▸, Table 1 ▸). Neighbouring complexes are additionally linked in the same direction by pairs of C—H⋯Cl hydrogen bonds between the chloro substituent of one complex and one pyridine H atom of a neighbouring complex (Fig. 2 ▸, Table 1 ▸). These chains are further linked by O—H⋯S hydrogen bonding between the second water H atom of one complex and a thio­cyanato S atom of a neighbouring complex into layers parallel to the *bc* plane (Fig. 3 ▸, Table 1 ▸). Within these layers, weak C—H⋯Cl hydrogen bonding is present (Table 1 ▸). Weak intra­molecular O—H⋯Cl inter­actions are also observed (Figs. 2 ▸ and 3 ▸, Table 1 ▸).

## Database survey   

To the best of our knowledge, there are only four coordination compounds containing thio­cyanato and 2-chloro­pyridine ligands deposited in the Cambridge Structure Database (Version 5.37, last update 2015; Groom *et al.*, 2016 ▸). The structures consist of tetra­hedrally coordinated metal cations (Co and Zn) where each metal cation is surrounded by two 2-chloro­pyridine ligands as well as two thio­cyanate anions (Wöhlert *et al.*, 2013 ▸). A general search for coordination compounds with 2-chloro­pyridine ligands resulted in 16 structures including the aforementioned ones. Two examples relate to a Pd compound, similar to the Co and Zn ones, however with the Pd^II^ cation in a square-planar conformation coordinated by two 2-chloro­pyridine ligands as well as two azide anions (Beck *et al.*, 2001 ▸) as well as a Cu compound with a square-pyramidal coordinated metal cation surrounded by two 2-chloro­pyridine ligands, one water ligand and two chloride anions (Jin *et al.*, 2005 ▸).

## Synthesis and crystallization   

Ba(NCS)_2_·3H_2_O, Ni(SO_4_)·6H_2_O and 2-chloro­pyridine were purchased from Alfa Aesar. Ni(NCS)_2_ was synthesized by stirring 17.5 g Ba(NCS)_2_·3H_2_O (57 mmol) with 15.0 g Ni(SO_4_)·6H_2_O (57 mmol) in 500 ml water. The green residue was filtered off and the filtrate was dried using a rotary evaporator. The homogeneity was checked by X-ray powder diffraction and elemental analysis. Crystals of the title compound suitable for single crystal X-ray diffraction were obtained by the reaction of 26.2 mg Ni(NCS)_2_ (0.15 mmol) with 56.0 µl 2-chloro­pyridine (0.6 mmol) in ethanol (1.0 ml) after a few days.

## Refinement   

Crystal data, data collection, and structure refinement details are summarized in Table 2 ▸. The CH H atoms were positioned with idealized geometry and were refined in a riding model with *U*_iso_(H) = 1.2*U*_eq_(C). The OH H atoms were located in a difference map, and their bond lengths constrained to 0.82 Å, with *U*_iso_(H) = 1.5*U*_eq_(O).

## Supplementary Material

Crystal structure: contains datablock(s) I. DOI: 10.1107/S2056989016015218/wm5326sup1.cif

Structure factors: contains datablock(s) I. DOI: 10.1107/S2056989016015218/wm5326Isup2.hkl

CCDC reference: 1506903

Additional supporting information: crystallographic information; 3D view; checkCIF report

## Figures and Tables

**Table 1:** Hydrogen-bond geometry (Å, °)

*D*—H⋯*A*	*D*—H	H⋯*A*	*D*⋯*A*	*D*—H⋯*A*
C11—H21⋯S1^i^	0.95	2.99	3.904 (4)	162
C13—H23⋯S1^ii^	0.95	2.99	3.796 (4)	143
C14—H24⋯Cl1^iii^	0.95	2.96	3.796 (4)	147
O1—H1*O*1⋯S1^iv^	0.82	2.39	3.175 (2)	160
O1—H2*O*1⋯S1^v^	0.82	2.53	3.239 (2)	145
O1—H2*O*1⋯Cl1^vi^	0.82	2.75	3.180 (3)	115

Symmetry codes: (i) 
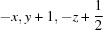
; (ii) 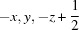; (iii) 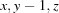; (iv) 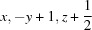; (v) 
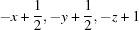
; (vi) 
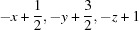
.

**Table 2:** Experimental details

Crystal data	
Chemical formula	[Ni(NCS)_2_(C_5_H_4_ClN)_2_(H_2_O)_2_]
*M*_r_	437.99
Crystal system, space group	Monoclinic, *C*2/*c*
Temperature (K)	200
*a*, *b*, *c* (Å)	19.5045 (15), 7.5486 (5), 14.9387 (11)
β (°)	125.560 (7)
*V* (Å^3^)	1789.3 (3)
*Z*	4
Radiation type	Mo *K*α
μ (mm^−1^)	1.63
Crystal size (mm)	0.14 × 0.09 × 0.06

Data collection	
Diffractometer	STOE *IPDS1*
Absorption correction	Numerical (*X-RED32* and *X-SHAPE*; Stoe, 2008 ▸)
*T*_min_, *T*_max_	0.796, 0.881
No. of measured, independent and observed [*I* > 2σ(*I*)] reflections	7195, 1568, 1321
*R*_int_	0.086
(sin θ/λ)_max_ (Å^−1^)	0.596

Refinement	
*R*[*F* ^2^ > 2σ(*F* ^2^)], *wR*(*F* ^2^), *S*	0.049, 0.123, 1.03
No. of reflections	1568
No. of parameters	107
H-atom treatment	H-atom parameters constrained
Δρ_max_, Δρ_min_ (e Å^−3^)	0.87, −0.87

Computer programs: *X-AREA* (Stoe, 2008 ▸), *SHELXS97* (Sheldrick, 2008 ▸), *SHELXL2014/7* (Sheldrick, 2015 ▸), *XP* in *SHELXTL* (Sheldrick, 2008 ▸), *DIAMOND* (Brandenburg, 1999 ▸) and *publCIF* (Westrip, 2010 ▸).

## supplementary crystallographic information

### Crystal data

**Table:**

[Ni(NCS)_2_(C_5_H_4_ClN)_2_(H_2_O)_2_]	*F*(000) = 888
*M**_r_* = 437.99	*D*_x_ = 1.626 Mg m^−^^3^
Monoclinic, *C*2/*c*	Mo *K*α radiation, λ = 0.71073 Å
*a* = 19.5045 (15) Å	Cell parameters from 7195 reflections
*b* = 7.5486 (5) Å	θ = 2.8–25.1°
*c* = 14.9387 (11) Å	µ = 1.63 mm^−^^1^
β = 125.560 (7)°	*T* = 200 K
*V* = 1789.3 (3) Å^3^	Block, blue
*Z* = 4	0.14 × 0.09 × 0.06 mm

### Data collection

**Table:**

STOE IPDS-1 diffractometer	1321 reflections with *I* > 2σ(*I*)
Phi scans	*R*_int_ = 0.086
Absorption correction: numerical (X-Red and X-Shape; Stoe, 2008)	θ_max_ = 25.1°, θ_min_ = 2.8°
*T*_min_ = 0.796, *T*_max_ = 0.881	*h* = −23→23
7195 measured reflections	*k* = −8→8
1568 independent reflections	*l* = −17→17

### Refinement

**Table:**

Refinement on *F*^2^	Hydrogen site location: mixed
Least-squares matrix: full	H-atom parameters constrained
*R*[*F*^2^ > 2σ(*F*^2^)] = 0.049	*w* = 1/[σ^2^(*F*_o_^2^) + (0.0887*P*)^2^] where *P* = (*F*_o_^2^ + 2*F*_c_^2^)/3
*wR*(*F*^2^) = 0.123	(Δ/σ)_max_ < 0.001
*S* = 1.03	Δρ_max_ = 0.87 e Å^−^^3^
1568 reflections	Δρ_min_ = −0.87 e Å^−^^3^
107 parameters	Extinction correction: SHELXL-2014/7 (Sheldrick 2015), Fc^*^=kFc[1+0.001xFc^2^λ^3^/sin(2θ)]^-1/4^
0 restraints	Extinction coefficient: 0.0046 (11)

### Special details

**Table:**

Geometry. All esds (except the esd in the dihedral angle between two l.s. planes) are estimated using the full covariance matrix. The cell esds are taken into account individually in the estimation of esds in distances, angles and torsion angles; correlations between esds in cell parameters are only used when they are defined by crystal symmetry. An approximate (isotropic) treatment of cell esds is used for estimating esds involving l.s. planes.

### Fractional atomic coordinates and isotropic or equivalent isotropic displacement parameters (Å^2^)

**Table:**

	*x*	*y*	*z*	*U*_iso_*/*U*_eq_	
Ni1	0.2500	0.7500	0.5000	0.0294 (3)	
N1	0.2189 (2)	0.5551 (4)	0.3900 (2)	0.0374 (7)	
C1	0.2063 (2)	0.4433 (4)	0.3291 (3)	0.0307 (7)	
S1	0.18836 (7)	0.28294 (11)	0.24339 (8)	0.0404 (3)	
N10	0.1155 (2)	0.7772 (4)	0.4334 (3)	0.0360 (7)	
C10	0.0670 (2)	0.9202 (4)	0.4075 (3)	0.0367 (8)	
C11	−0.0154 (3)	0.9163 (6)	0.3732 (3)	0.0500 (10)	
H21	−0.0466	1.0227	0.3568	0.060*	
C12	−0.0520 (3)	0.7528 (6)	0.3630 (4)	0.0586 (12)	
H22	−0.1088	0.7447	0.3395	0.070*	
C13	−0.0039 (3)	0.6027 (6)	0.3879 (4)	0.0520 (10)	
H23	−0.0271	0.4888	0.3814	0.062*	
C14	0.0780 (3)	0.6201 (5)	0.4222 (3)	0.0425 (8)	
H24	0.1104	0.5153	0.4391	0.051*	
Cl1	0.11007 (7)	1.12552 (11)	0.41519 (9)	0.0523 (4)	
O1	0.26098 (19)	0.5775 (3)	0.6135 (2)	0.0506 (8)	
H1O1	0.2506	0.5933	0.6588	0.076*	
H2O1	0.2750	0.4730	0.6262	0.076*	

### Atomic displacement parameters (Å^2^)

**Table:**

	*U*^11^	*U*^22^	*U*^33^	*U*^12^	*U*^13^	*U*^23^
Ni1	0.0383 (4)	0.0205 (4)	0.0375 (4)	0.0053 (2)	0.0267 (3)	0.0031 (2)
N1	0.0443 (18)	0.0298 (14)	0.0434 (17)	0.0060 (11)	0.0285 (15)	−0.0008 (12)
C1	0.0350 (18)	0.0247 (15)	0.0417 (19)	0.0052 (12)	0.0276 (17)	0.0077 (13)
S1	0.0613 (7)	0.0251 (4)	0.0505 (6)	−0.0030 (3)	0.0415 (5)	−0.0032 (3)
N10	0.0388 (17)	0.0317 (14)	0.0423 (17)	0.0040 (11)	0.0264 (15)	0.0018 (11)
C10	0.036 (2)	0.0375 (17)	0.0383 (19)	0.0087 (14)	0.0225 (17)	0.0037 (14)
C11	0.036 (2)	0.064 (2)	0.043 (2)	0.0151 (18)	0.019 (2)	0.0064 (18)
C12	0.034 (2)	0.087 (4)	0.050 (3)	−0.0007 (19)	0.022 (2)	−0.004 (2)
C13	0.042 (2)	0.057 (2)	0.054 (3)	−0.0120 (19)	0.026 (2)	−0.0078 (19)
C14	0.042 (2)	0.0371 (18)	0.051 (2)	−0.0066 (14)	0.029 (2)	−0.0054 (15)
Cl1	0.0574 (7)	0.0308 (5)	0.0770 (8)	0.0163 (4)	0.0438 (6)	0.0128 (4)
O1	0.079 (2)	0.0367 (13)	0.0661 (18)	0.0270 (13)	0.0597 (18)	0.0243 (12)

### Geometric parameters (Å, º)

**Table:**

Ni1—N1^i^	2.018 (3)	C10—Cl1	1.734 (4)
Ni1—N1	2.018 (3)	C11—C12	1.389 (6)
Ni1—O1	2.048 (2)	C11—H21	0.9500
Ni1—O1^i^	2.048 (2)	C12—C13	1.377 (6)
Ni1—N10	2.208 (3)	C12—H22	0.9500
Ni1—N10^i^	2.208 (3)	C13—C14	1.372 (6)
N1—C1	1.158 (4)	C13—H23	0.9500
C1—S1	1.645 (3)	C14—H24	0.9500
N10—C10	1.336 (4)	O1—H1O1	0.8198
N10—C14	1.352 (4)	O1—H2O1	0.8201
C10—C11	1.375 (6)		
			
N1^i^—Ni1—N1	180.0	C14—N10—Ni1	112.9 (2)
N1^i^—Ni1—O1	87.27 (12)	N10—C10—C11	124.6 (4)
N1—Ni1—O1	92.73 (12)	N10—C10—Cl1	117.9 (3)
N1^i^—Ni1—O1^i^	92.73 (12)	C11—C10—Cl1	117.4 (3)
N1—Ni1—O1^i^	87.27 (12)	C10—C11—C12	118.3 (4)
O1—Ni1—O1^i^	180.0	C10—C11—H21	120.8
N1^i^—Ni1—N10	90.88 (11)	C12—C11—H21	120.8
N1—Ni1—N10	89.12 (11)	C13—C12—C11	118.4 (4)
O1—Ni1—N10	87.55 (11)	C13—C12—H22	120.8
O1^i^—Ni1—N10	92.45 (11)	C11—C12—H22	120.8
N1^i^—Ni1—N10^i^	89.11 (11)	C14—C13—C12	119.0 (4)
N1—Ni1—N10^i^	90.88 (11)	C14—C13—H23	120.5
O1—Ni1—N10^i^	92.45 (11)	C12—C13—H23	120.5
O1^i^—Ni1—N10^i^	87.55 (11)	N10—C14—C13	124.0 (4)
N10—Ni1—N10^i^	180.0	N10—C14—H24	118.0
C1—N1—Ni1	175.7 (3)	C13—C14—H24	118.0
N1—C1—S1	179.4 (3)	Ni1—O1—H1O1	129.3
C10—N10—C14	115.6 (3)	Ni1—O1—H2O1	131.5
C10—N10—Ni1	131.4 (2)	H1O1—O1—H2O1	99.1

Symmetry code: (i) −*x*+1/2, −*y*+3/2, −*z*+1.

### Hydrogen-bond geometry (Å, º)

**Table:**

*D*—H···*A*	*D*—H	H···*A*	*D*···*A*	*D*—H···*A*
C11—H21···S1^ii^	0.95	2.99	3.904 (4)	162
C13—H23···S1^iii^	0.95	2.99	3.796 (4)	143
C14—H24···Cl1^iv^	0.95	2.96	3.796 (4)	147
O1—H1*O*1···S1^v^	0.82	2.39	3.175 (2)	160
O1—H2*O*1···S1^vi^	0.82	2.53	3.239 (2)	145
O1—H2*O*1···Cl1^i^	0.82	2.75	3.180 (3)	115

Symmetry codes: (i) −*x*+1/2, −*y*+3/2, −*z*+1; (ii) −*x*, *y*+1, −*z*+1/2; (iii) −*x*, *y*, −*z*+1/2; (iv) *x*, *y*−1, *z*; (v) *x*, −*y*+1, *z*+1/2; (vi) −*x*+1/2, −*y*+1/2, −*z*+1.
